# Evaluation of a novel immunoassay to detect p-tau Thr217 in the CSF to distinguish Alzheimer disease from other dementias

**DOI:** 10.1212/WNL.0000000000010814

**Published:** 2020-12-01

**Authors:** Jozef Hanes, Andrej Kovac, Hlin Kvartsberg, Eva Kontsekova, Lubica Fialova, Stanislav Katina, Branislav Kovacech, Eva Stevens, Jakub Hort, Martin Vyhnalek, Lynn Boonkamp, Michal Novak, Henrik Zetterberg, Oskar Hansson, Philip Scheltens, Kaj Blennow, Charlotte E. Teunissen, Norbert Zilka

**Affiliations:** From the AXON Neuroscience R&D Services SE (J. Hanes, A.K., E.K., L.F., B.K., E.S., N.Z.), Bratislava, Slovakia; Department of Psychiatry and Neurochemistry (H.K., H.Z., K.B.), Institute of Neuroscience and Physiology, the Sahlgrenska Academy at the University of Gothenburg, Mölndal; Clinical Neurochemistry Laboratory (H.K., H.Z., K.B.), Sahlgrenska University Hospital, Mölndal, Sweden; AXON Neuroscience CRM Services SE (S.K.), Bratislava, Slovakia; International Clinical Research Centre (J. Hort, M.V.), St. Anne's University Hospital Brno; Memory Clinic, Department of Neurology (J. Hort, M.V.), Charles University, 2nd Faculty of Medicine and Motol University Hospital, Czech Republic; Department of Clinical Chemistry, Neurochemistry Laboratory (L.B., C.E.T.), Amsterdam Neuroscience, VU University Medical Center Amsterdam, the Netherlands; Axon Neuroscience SE (M.N.), Larnaca, Cyprus; Department of Neurodegenerative Disease (H.Z.), UCL Institute of Neurology, Queen Square, London; UK Dementia Research Institute at UCL (H.Z.), London; Clinical Memory Research Unit (O.H.), Department of Clinical Sciences Malmö, Lund University; Memory Clinic (O.H.), Skåne University Hospital, Malmö, Sweden; and Department of Neurology, Alzheimer Center (P.S.), Amsterdam Neuroscience, the Netherlands.

## Abstract

**Objective:**

To investigate whether tau phosphorylated at Thr217 (p-tau T217) assay in CSF can distinguish patients with Alzheimer disease (AD) from patients with other dementias and healthy controls.

**Methods:**

We developed and validated a novel Simoa immunoassay to detect p-tau T217 in CSF. There was a total of 190 participants from 3 cohorts with AD (n = 77) and other neurodegenerative diseases (n = 69) as well as healthy participants (n = 44).

**Results:**

The p-tau T217 assay (cutoff 242 pg/mL) identified patients with AD with accuracy of 90%, with 78% positive predictive value (PPV), 97% negative predictive value (NPV), 93% sensitivity, and 88% specificity, compared favorably with p-tau T181 ELISA (52 pg/mL), showing 78% accuracy, 58% PPV, 98% NPV, 71% specificity, and 97% sensitivity. The assay distinguished patients with AD from age-matched healthy controls (cutoff 163 pg/mL, 98% sensitivity, 93% specificity), similarly to p-tau T181 ELISA (cutoff 60 pg/mL, 96% sensitivity, 86% specificity). In patients with AD, we found a strong correlation between p-tau T217 and p-tau T181, total tau and β-amyloid 40, but not β-amyloid 42.

**Conclusions:**

This study demonstrates that p-tau T217 displayed better diagnostic accuracy than p-tau T181. The data suggest that the new p-tau T217 assay has potential as an AD diagnostic test in clinical evaluation.

**Classification of evidence:**

This study provides Class III evidence that a CSF immunoassay for p-tau T217 distinguishes patients with AD from patients with other dementias and healthy controls.

In Alzheimer disease (AD), there is a need for biomarkers that reflect the key pathophysiology of the disease: neurodegeneration and β-amyloid (Aβ) and tau protein pathology.^1^ Over the past 2 decades, significant efforts have been made to identify in vivo brain indicators and fluid-based biomarkers for preclinical and clinical AD.^2–4^

In 2018, the National Institute on Aging and Alzheimer's Association Research Framework shifted the definition of AD in living people from a syndromal to a biological construct.^5^ The new research framework defines AD by using a variety of biomarkers, which are grouped into those of Aβ deposition, pathologic tau protein, and neurodegeneration (A/T/N).^5^

Recently, a new biomarker for AD has been reported: tau phosphorylated at Thr217 (p-tau T217). It has been shown that p-tau T217 species (quantified as pT217/T217 ratio) highly correlate with amyloid lesions in the brain, cognitive decline, and tau PET imaging in AD.^6–8^ In physiologic conditions, p-tau T217 species display rapid turnover in the extracellular space.^8^

We developed a novel immunoassay to detect p-tau T217 in the CSF, and subsequently undertook this study to (1) evaluate the sensitivity and specificity of the p-tau T217 assay using AD and non-AD CSF samples from 3 international cohorts of patients; (2) compare its sensitivity and specificity with standard CSF measures (particularly with total tau [t-tau], p-tau T181, Aβ40, and Aβ42); and (3) assess the relationship between the levels of p-tau T217 with the above-mentioned standard CSF biomarkers.

## Methods

### Standard protocol approvals, registrations, and participant consents

Written informed consent was obtained from all patients (or guardians of patients) participating in the study. All protocols were approved by the ethical committees of Alzheimer Center, Amsterdam Neuroscience, Amsterdam, the Netherlands; Lund University, Sweden; or Motol University Hospital, Prague, Czech Republic.

### Assay development and validation

#### Preparation of hybridoma cell lines expressing DC2E7 and DC2E2 antibodies and their purification

We prepared DC2E7 and DC2E2 hybridoma cell lines as described previously.^9^ To generate the DC2E7 and DC2E2 antibodies, we immunized Balb/c mice with either sarkosyl-insoluble tau (PHF-tau) isolated from AD brain (frontal cortex, Braak stage VI, Netherlands brain bank) or recombinant human tau protein (aa 1–242 of the longest tau isoform). Antibodies were purified using protein G affinity chromatography by Äkta Avant Purifier (both GE Healthcare, Chicago, IL).

Analyzing of both antibodies showed that DC2E7 recognizes p-tau protein, while DC2E2 is a pan-tau antibody recognizing a proline-rich domain of tau protein. To define the exact phosphoepitope for DC2E7, we generated mutated forms of tau 2N4R with single point mutations in which serine and threonine residues were replaced by alanine. The immunoblotting analysis showed that antibody DC2E7 recognized all p-tau proteins carrying the point mutations except for Thr217Ala. This suggests that phospho-threonine at position 217 creates a key part of the epitope recognized by antibody DC2E7 (supplemental figure 1, data available from Dryad, doi.org/10.5061/dryad.tdz08kpwz).

### p-tau T217 Simoa assay

We set up the p-tau T217 assay in the highly sensitive format of a single molecule array (Simoa) digital ELISA, using an HD-1 Analyzer (Quanterix, Billerica, MA). Reagents for the assay were prepared according to the Quanterix Homebrew Assay Development Guide with the following details: we used DC2E7 antibody as a capture antibody and DC2E2 antibody as a detector antibody; the capture antibody DC2E7 was coupled to magnetic beads (Quanterix) at a concentration of 0.5 mg/mL according to the Simoa alternate bead conjugation protocol (2017); the detector antibody DC2E2 was prepared by biotinylation of DC2E2, where 120-fold excess of biotin, EZ‐Link NHS‐PEG4‐Biotin (Thermo Fisher Scientific, Waltham, MA; 21329), over antibody concentration was used.

We prepared the pT217 calibrator as a synthetic peptide containing both epitopes of the DC2E7 and DC2E2 antibodies. This peptide was dissolved in a calibrator diluent at a concentration of 2 µg/mL, aliquoted and stored at −80°C. The calibrator was diluted in a calibrator diluent (20 mM sodium phosphate pH 7.4, 137 mM NaCl, 2.7 mM KCl, 2% bovine serum albumin) in serial 1.6-fold dilutions starting from 2,000 pg/mL, followed by 1,250, 781.25, 488.28, 305.18, 190.73, 119.21, 74.51, and 0 pg/mL. The prepared calibrator concentrations were mixed in a 3:1 ratio with a sample diluent (80 mM sodium phosphate pH 7.4, 548 mM NaCl, 10.8 mM KCl, 0.04% casein, and 0.4% Tween 20). The CSF samples were diluted with a sample diluent in the same way as described for the calibrator (3 volumes CSF +1 volume sample diluent). The assay was performed as a 2-step assay 1.0 according to the manufacturer's recommendation (Quanterix).

### Validation of the assay: experimental setup

The validation was conducted in an open-label fashion at the Department of Clinical Chemistry, Amsterdam, the Netherlands. The protocol for p-tau T217 detection and quantitation was applied to Simoa using the HD-1 Analyzer. The assay's sensitivity, precision, linearity, parallelism, and recovery were analyzed as previously described.^10^ To identify the lower limit of quantification (LLOQ), 16 blank samples (240 μL calibrator diluent + 80 μL sample diluent) were measured. The LLOQ (concentration) was based on a signal of 10 SDs above the mean of the 16 blank samples (using the calibration curve). Precision was determined by calculating the intra-assay, interassay, and intraplate reproducibility. For the dilution linearity, 3 different CSF samples were spiked with 3,000 pg/mL of pT217 calibrator and diluted 2-fold until below the theoretical LLOQ. To assess parallelism, 5 different CSF samples were diluted 2-fold. To determine sample recovery, 4 different CSF samples were spiked with low (200 pg/mL), medium (500 pg/mL), or high (1,500 pg/mL) concentrations of the pT217 calibrator. Phosphate-buffered saline was spiked as a reference.

## Clinical cohorts

### Participants

The first cohort from the ongoing Amsterdam Dementia Cohort included 88 participants with age-matched controls: AD, nonfluent variant of primary progressive aphasia (nvPPA), semantic variant of primary progressive aphasia (svPPA), behavioral variant of frontotemporal dementia (bvFTD), progressive supranuclear palsy (PSP), and corticobasal degeneration (CBD) (table 1). All participants visited the memory clinic at VU University Medical Center Amsterdam for extensive clinical evaluations that consisted of neurologic, physical, and neuropsychological evaluations, biomarker analyses in CSF, EEG, and brain MRI.^11^

**Table 1:**
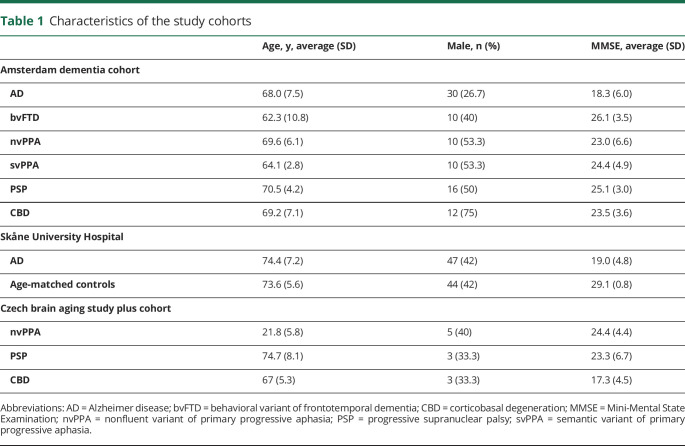
Characteristics of the study cohorts

The second cohort included 44 cognitively normal elderly participants and 47 patients with AD recruited from the Skåne University Hospital, Sweden. The inclusion criteria for the cognitively normal elderly participants were age ≥60 years, a Mini-Mental State Examination score of 28–30 points at the screening visit, absence of cognitive symptoms as evaluated by a physician, fluency in Swedish, and not fulfilling the criteria of mild cognitive impairment or any dementia. Patients with AD were required to meet the criteria for probable AD as defined by the National Institute of Neurologic and Communicative Disorders and Stroke–Alzheimer's Disease and Related Disorders Association.^12^

The third cohort included patients from the Czech Brain Aging Study Plus Cohort. In total, 11 patients with probable non-AD tauopathy were included: nvPPA (n = 5), PSP (n = 3), and CBD (n = 3). Patients with nvPPA fulfilled imaging criteria for nvPPA as described by Gorno-Tempini et al.^13^ Patients with PSP were diagnosed with probable PSP with Richardson syndrome^14^ and patients with CBD fulfilled the criteria for probable sporadic CBS.^15^

### CSF sampling and core AD CSF biomarkers

CSF was handled in compliance with standard recommendations.^16^ All the samples were collected and stored in polypropylene tubes at −80°C according to lumbar puncture consensus protocols.^17^ Aβ42, t-tau, p-tau T181, and Aβ40 were measured by Fujirebio (Tokyo, Japan) Innotest ELISA assays. For the analysis of Aβx-42/Aβx-40 ratio in cohort 2, a Meso Scale Discovery (Rockville, MD) Abeta Triplex assay was used. Cohorts 1 and 3 were analyzed in Amsterdam and cohort 2 was analyzed in Gothenburg. All analyses were conducted in an open-label fashion.

### Immunohistochemical staining

For immunohistochemistry, the following brain areas were used: hippocampus and entorhinal cortex from AD (Braak stage VI, n = 3), frontotemporal dementia (FTD) (Pick disease, n = 3), control brain (Braak stage I, n = 3), and prodromal AD (Braak stage III, n = 3); caudate nucleus from CBD (n = 3); and putamen/caudate nucleus from PSP (n = 3). The brain tissue paraffin blocks were obtained from the Amsterdam brain bank.

The brain blocks embedded in paraffin were cut on a microtome (Leica [Newcastle, UK] RM2255) to obtain 8-μm-thick sections. The sections were placed on HistoBond slides (Marienfeld, Germany). Immunohistochemistry sections were pretreated with formic acid and heat (autoclave, 121°C, 20 minutes), followed by overnight incubation with primary antibodies (AT8 1:1,000, DC2E7 1:10,000, DC2E2 1:200). All sections were incubated with anti-mouse biotinylated secondary antibody at room temperature for 1 hour and with avidin-biotin peroxidase complex for 1 hour. The immunoreaction was visualized with VIP (Vectastain Elite ABC Kit; Vector Laboratories, Burlingame, CA) and counterstained with methyl green (Vector Laboratories).

### Statistical analysis

First, the performance of the diagnostic assay was evaluated based on diagnostic accuracy, positive predictive value, negative predictive value, sensitivity, and specificity. False-positive fraction (1 − specificity) and true-positive fraction (sensitivity) were calculated. Based on this result, area under the curve (AUC), 95% confidence interval (CI) for AUC, and optimal threshold were calculated.^18^ Where necessary, a linear approximation to calculate specificity and sensitivity for prespecified thresholds was used. The equality of AUC curves using the 2‐sample Wald *Z* test was also tested.^19^ Second, the differences in means of p-tau T217 between AD and other diagnoses were assessed using a bootstrap 2-sample *t* test (the number of bootstrap samples was 10,000) followed by Bonferroni multiple adjustment of *p* values. The differences in means of p-tau T217 between patients with AD and controls were assessed using a bootstrap 2-sample *t* test (number of bootstrap samples was 10,000). The effect size Cohen *d* and its empirical Wald 95% CI was calculated using variance stabilizing transformation.^20^ Finally, the association between various CSF biomarkers was characterized by Pearson product-moment correlation coefficient and tested using Fisher *Z* test.^21^ All alternative hypotheses were 2-sided and statistical tests were performed at significance level equal to 0.05.

### Primary research question

Does the p-tau T217 assay distinguish AD dementia from other dementias and healthy controls? This study provides Class III evidence that a CSF immunoassay for p-tau T217 distinguishes AD from other dementias and healthy controls.

### Data availability

Anonymized data will be shared on request from qualified investigators.

## Results

### Validation of the p-tau T217 ultrasensitive immunoassay for human CSF

LLOQs based on 16 blank values were calculated to be 184.4 pg/mL. The mean parallelism of all 5 samples was 86%. Two out of 5 CSF samples fell within the acceptable range (85%–115%) and were thus parallel to the pT217 calibrator. The other 3 CSF samples were just outside the criteria of 85%–115% (81%, 82%, and 76%). The mean recovery of 3 CSF samples was 116% (low 110%, medium 116%, high 123%), just outside the predefined criteria of 85%–115%, meaning that there was almost no difference between the sample matrix and calibrator diluent. The assay was linear between a 2- and 16-fold dilution and no hook effect was observed. The precision of the assay was determined by means of the repeatability (intra-assay; 3.0%), intermediate precision (interassay; 10.3%), and within-plate reproducibility (intraplate; 3.4%) (supplemental table 1, data available from Dryad, doi.org/10.5061/dryad.tdz08kpwz). The results showed a robust performance of the assay. Overall, we concluded that the assay for p-tau T217 on the Simoa HD-1 Analyzer is suitable for the measurement of p-tau T217 in human CSF samples.

### Monoclonal antibodies DC2E2 and DC2E7 recognize pathology in human AD brain and tauopathies

**Figure 1:**
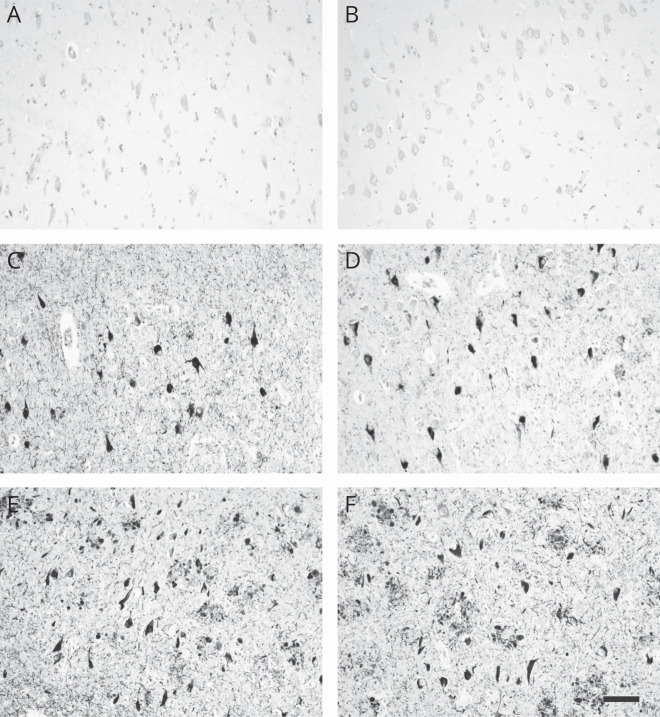
Monoclonal antibodies pathology recognition in normal brain, prodromal stage, and Alzheimer disease (AD) Monoclonal antibodies DC2E2 and DC2E7 do not recognize tau in a normal brain (A and B, Braak stage I). In the prodromal stage (C and D, Braak stage III) and full-blown AD (E and F, Braak stage VI), both antibodies identify neurofibrillary pathology. They stain neurofibrillary tangles, neuropil threads, and neuritic plaques. Bar = 100 µm.

Neither DC2E2 nor DC2E7 recognized normal tau in the hippocampus of healthy controls fulfilling criteria for Braak stage I (figure 1A). In patients in the prodromal stage of AD (Braak stage III), the antibodies identified neurofibrillary tangles, neuropil threads, and neuritic plaques distributed mainly in the hippocampus and entorhinal and transentorhinal cortex (figure 1C). Finally, in late stage AD (Braak stage VI), the antibodies recognized extensive tau pathology in the hippocampus (figure 1E) and other cortical areas.

**Figure 2:**
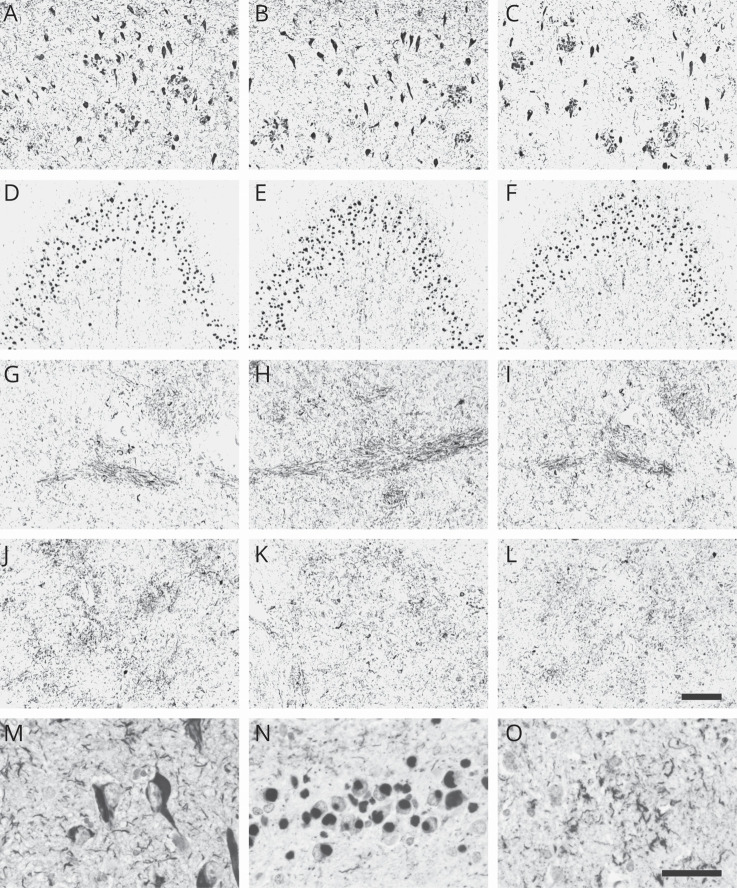
Monoclonal antibodies pathology recognition in Alzheimer disease (AD), frontotemporal dementia (FTD), and corticobasal degeneration (CBD) or progressive supranuclear palsy (PSP) Monoclonal antibodies DC2E7 and DC2E2 recognized neurofibrillary pathology in the hippocampus of patients with AD (A and B), Pick bodies in the dentate gyrus of patients with FTD (D and E), and glial tau pathology in caudate nucleus of patients with CBD (G and H) or PSP (J and K). AT8 was used as a control for histopathologic staining (C, F, I, and L). Higher magnification of tau pathology in AD (M), Pick disease (N), and CBD (O). Bar = 200 µm (A–L), 50 µm (M–O).

In addition to AD (figure 2, A and B; higher magnification M), both DC2E7 and DC2E2 recognized tau pathology in other tauopathies, including Pick disease, CBD, and PSP (figure 2; D and E, higher magnification N; G and H, higher magnification O; J and K). The antibodies displayed the same staining pattern and the same type and load of tau pathology as monoclonal antibody AT8, which is considered to be the gold standard for histopathologic staining^22^ (figure 2, C, F, I, and L).

### The p-tau T217 ultrasensitive immunoassay differentiates AD from FTD

**Figure 3:**
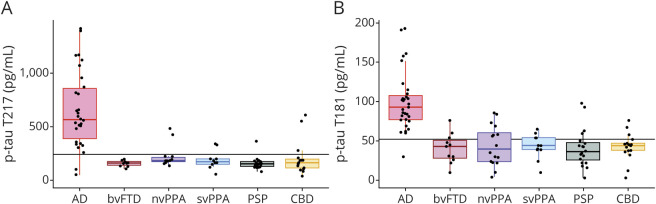
CSF phosphorylated tau (p-tau) T217 and p-tau T181 levels CSF p-tau T217 (A) and p-tau T181 (B) levels in Alzheimer disease (AD), behavioral variant of frontotemporal dementia (bvFTD), nonfluent variant of primary progressive aphasia (nvPPA), semantic variant of primary progressive aphasia (svFTD), progressive supranuclear palsy (PSP), and corticobasal degeneration (CBD) The lines indicate cutoff values (p-tau T217, 242 pg/mL; p-tau T181, 184 pg/mL). CSF samples obtained from cohorts 1 and 3.

We measured p-tau T217 concentration in CSF from patients with nvPPA, svPPA, bvFTD, PSP, and CBD (cohorts 1 and 3; figure 3A). The p-tau T217 assay discriminated between AD and non-AD neurodegenerative disorders (cutoff 242 pg/mL, AUC 0.91 [95% CI 0.80, 0.96], with accuracy of 90%, with 78% PPV, 97% NPV, 88% specificity [95% CI 0.79, 0.98], and 93% sensitivity [95% CI 0.78, 0.99]) compared to p-tau T181 ELISA (figure 3B) (cutoff 52 pg/mL, AUC 0.94 [95% CI 0.84, 0.98], showing 78% accuracy, 58% PPV, 98% NPV, 71% specificity [95% CI 0.44, 0.99], and 97% sensitivity [95% CI 0.89, 0.99]). There was no statistically significant difference in AUCs between p-tau T217 and p-tau T181 (*p* value = 0.912) (supplemental figure 2, data available from Dryad, doi.org/10.5061/dryad.tdz08kpwz).

Comparison of means showed that the assay significantly differentiated AD and nvPPA (*p* < 0.0001; Cohen *d* 1.433, 95% CI 0.758, 2.171), svPPA (*p* < 0.0001; Cohen *d* 1.469, 95% CI 0.714, 2.296), bvFTD (*p* < 0.0001; Cohen *d* 1.586, 95% CI 0.822, 2.426), PSP (*p* < 0.0001; Cohen *d* 1.745, 95% CI 1.105, 2.455), and CBD (*p* < 0.0001, Cohen *d* 1.452; 95% CI 0.79, 2.177).

### The p-tau T217 ultrasensitive immunoassay differentiates AD from controls

**Figure 4:**
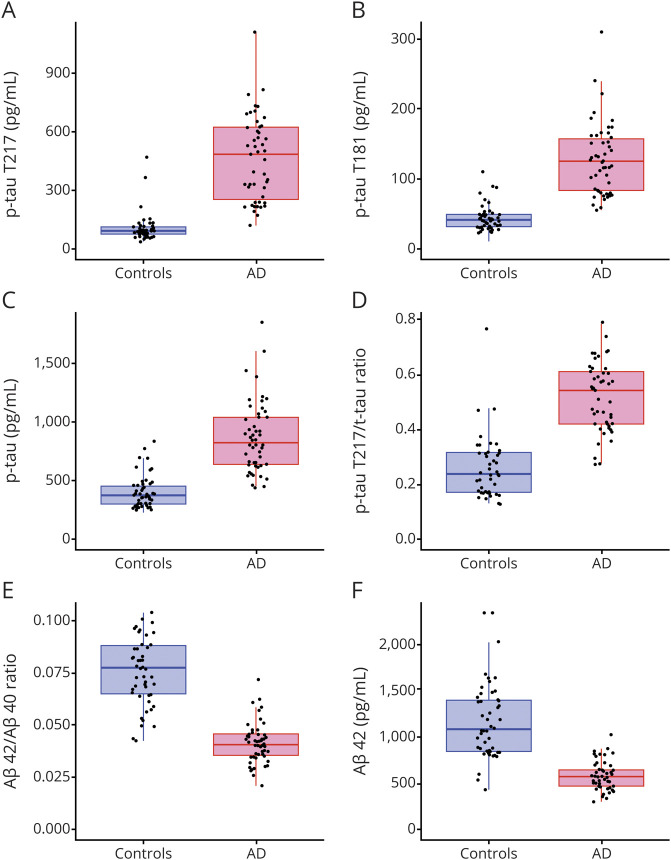
Phosphorylated tau (p-tau) T217, p-tau T181, total tau (t-tau), and β-amyloid (Aβ) levels Levels of p-tau T217 (A), p-tau T181 (B), t-tau (C), ratio p-tau T217/t-tau (D), Aβ42 (E), and ratio Aβ42/40 (F) in Alzheimer disease (AD) and controls. CSF samples obtained from cohort 2.

Further, we aimed to prove the diagnostic value of the assay to distinguish individuals with AD from healthy individuals. The p-tau T217 immunoassay was used to analyze CSF samples from patients with AD (n = 47) and control individuals (n = 44) (cohort 2). We found that the assay distinguished patients with AD from healthy individuals with very high sensitivity and specificity (cutoff 162.8 pg/mL, AUC 0.98 [95% CI 0.91, 0.99], sensitivity 98% [95% CI 0.88, 0.99], specificity 93% [95% CI 0.85, 0.99]; figure 4A). In comparison, p-tau T181 ELISA (cutoff 60 pg/mL, AUC 0.98 [95% CI 0.91, 0.99]), currently considered one of the best biomarkers for AD, showed 98% sensitivity (95% CI 0.88,0.99) and 86% specificity (95% CI 0.76, 0.99) (figure 4B). There was no statistically significant difference in AUCs between p-tau T217 and p-tau T181 (*p* = 0.574). Mean comparison showed that the p-tau T217 assay significantly differentiated between AD and controls (*p* < 0.0001; Cohen *d* 2.160, 95% CI 1.663, 2.701). Other commonly used CSF biomarkers for AD—t-tau, Aβ42, Aβ42/Aβ40 ratio, and p-tau T217/t-tau ratio—showed worse diagnostic performance than p-tau pT217 alone (figure 4, C–F).

### The amount of p-tau T217 correlates with that of p-tau T181, t-tau, and Aβ40 but not with Aβ42

**Figure 5:**
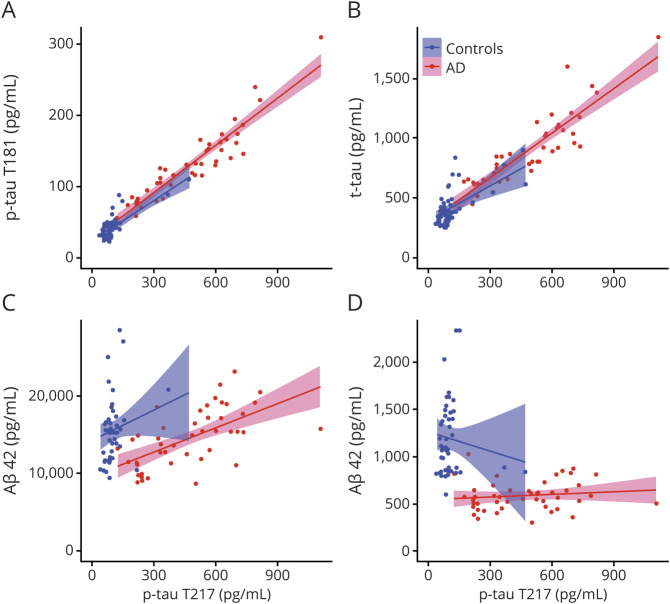
Correlations between phosphorylated tau (p-tau) T217 and p-tau T181, total tau (t-tau), and β-amyloid (Aβ) Correlation between p-tau T217 and p-tau T181 (A), t-tau (B), Aβ40 (C), and Aβ42 (D) in Alzheimer disease (AD) and controls. CSF samples obtained from cohort 2.

In the second cohort, we found a strong correlation between p-tau T217 and p-tau T181 (figure 5A, *r* = 0.941, 95% CI 0.896, 0.967, *p* < 0.0001) and between p-tau T217 and t-tau (figure 5B, *r* = 0.902, 95% CI 0.829, 0.944, *p* < 0.0001) in AD. Interestingly, we observed a correlation between p-tau T217 and Aβ40 (figure 5C, *r* = 0.617, 95% CI 0.402, 0.768, *p* < 0.0001) but not between p-tau T217 and Aβ42 (figure 5D, *r* = 0.131, 95% CI −0.162, 0.403, *p* = 0.380) in AD. The correlation between p-tau T217 and other tau CSF biomarkers was weaker in healthy individuals: between p-tau T217 and p-tau T181 (figure 5A, *r* = 0.787, 95% CI 0.640, 0.879, *p* < 0.0001), and between p-tau T217 and t-tau (figure 5B, *r* = 0.541, 95% CI 0.290, 0.722, *p* = 0.0001). We did not find any correlations between p-tau T217 and Aβ40 (figure 5C, *r* = 0.235, 95% CI −0.066, 0.497, *p* = 0.125) or between p-tau T217 and Aβ42 (figure 5D, *r* = −0.126, 95% CI −0.407, 0.178, *p* = 0.419) in healthy individuals.

## Discussion

Previous studies have demonstrated that CSF p-tau T217 could potentially be a key biomarker to monitor tau pathology in AD pathophysiology, and that its role might differ from other p-tau biomarkers.^6,7^ In this study, we introduced a novel immunoassay, detecting p-tau T217 in CSF, which is based on the highly sensitive Simoa technology. Our results show that the assay discriminates between AD and other neurodegenerative dementias with high specificity and sensitivity, and demonstrates better diagnostic accuracy than p-tau T181 assay. This is in line with recent study on the new CSF p-tau T217 assay based on the MSD (Meso Scale Discovery) platform, where the authors showed better diagnostic performance of the assay when comparing with p-tau T181.^23^ Moreover, the same study revealed that the correlations with tau PET tracer [^18^F]flortaucipir were consistently higher for p-tau217 than p-tau181 and that [^18^F]flortaucipir retention was more related to longitudinal changes in p-tau217 than in p-tau181. Similarly, by using the quantitative mass spectrometry approach, Barthelemy et al.^24^ demonstrated that pT217 differentiated between AD and other neurodegenerative diseases with higher specificity and sensitivity than pT181. The better discriminatory potency of p-tau T217 for AD might be attributed to its ability to reflect both amyloid and tau pathologic pathways. It has been reported that increased p-tau T217 levels in CSF are related to the brain amyloid load–positive participants already at the preclinical stage, further supporting that this biomarker is AD-specific.^6^ The specificity of T217 tau phosphorylation change for AD surpasses other p-tau sites such as T181, S199, S202, and T205.^6^ These data suggest that p-tau T217 represents a promising AD biomarker.

The assay also distinguishes patients with AD from age-matched healthy controls, which is in agreement with mass spectrometry data on p-tau T217.^24^ Low levels of p-tau T217 in healthy controls could be caused by rapid degradation (or dephosphorylation) of tau species phosphorylated at this particular position. Indeed, one study has demonstrated that phosphorylation of tau on T217 had the most robust effect on shortening tau half-life in physiologic conditions.^8^

When investigating the relationship between the different biomarkers in patients with AD, p-tau T217 showed a strong correlation with t-tau, p-tau T181, and Aβ40, but not with Aβ42. It was hypothesized that Aβ42 is toxic to neurons, while Aβ40 is more strongly associated with progressive neuronal degeneration.^25^ The strong correlation between p-tau T217, t-tau, p-tau T181, and Aβ40 suggests that these proteins may be released from neurons in a coordinated fashion, perhaps in relation to neuronal activity, as has been suggested.^26,27^

Interestingly, our p-tau T217 assay discriminates AD from FTD, despite the fact that p-tau T217 was present in brain tissues of both AD and FTD. The p-tau T217 species were found to be present in all types of neurofibrillary lesions (neurofibrillary tangles, neuropil threads, dystrophic neurites, and neuritic plaques), but also in glial tau pathology (PSP, CBD) and in Pick bodies (FTD). Although our findings indicate that p-tau T217 species are involved in the developing tau pathology in neurons and glial cells in various human tauopathies, the CSF levels of p-tau T217 species are elevated almost exclusively in AD.

The potential limitation of our study could be the small sample size. Results should be replicated in larger cohorts ideally characterized by both amyloid and tau PET imaging and validated in routine clinical practice. In order to introduce p-tau T217 assay in clinical routine practice, the technology should undergo a structured assessment to evaluate its benefit in terms of clinical utility and cost-effectiveness.

Our novel immunoassay for quantification of p-tau T217 in the CSF demonstrates that the assay is highly specific for AD and seems superior to the p-tau T181 assay in AD diagnostic classifications. In the future, the assay can potentially be used for diagnostic purposes as well as for patient stratification and enrichment of target populations in clinical trials for disease-modifying therapies.

## Glossary

Aβ: β-amyloid
AD: Alzheimer disease
AUC: area under the curve
bvFTD: behavioral variant of frontotemporal dementia
CBD: corticobasal degeneration
CI: confidence interval
FTD: frontotemporal dementia
LLOQ: lower limit of quantification
nvPPA: nonfluent variant of primary progressive aphasia
p-tau: phosphorylated tau
PSP: progressive supranuclear palsy
svPPA: semantic variant of primary progressive aphasia
t-tau: total tau
